# Bibliometric Analysis of the 100 Most-Cited Publications in Gender-Affirming Surgery

**DOI:** 10.1093/asjof/ojag020

**Published:** 2026-02-04

**Authors:** Nir Zontag, Ron Skorochod, Yoram Wolf

## Abstract

Gender-affirming surgeries (GASs) serve as a fundamental treatment for gender dysphoria, with growing evidence of their benefits in both health outcomes and quality of life. The aim of this study was to identify and analyze the 100 most-cited articles in the field, to highlight impactful studies, research trends, and prominent authors. A search of the Web of Science database was conducted to identify the top 100 most-cited articles related to GAS. Bibliometric and visual analyses were performed using VOSviewer, Bibliometrix, and Biblioshiny packages for R statistical software. The authors gathered and analyzed citation metrics, institutional networks, collaboration clusters, thematic keyword analysis, and author demographics. The 100 most-cited articles on GAS were published between 1980 and 2023, with an increase in both publication and citations after 2015. The thematic analysis demonstrated 2 primary research domains which focused on patient quality of life and surgical outcomes. The research institutions Vrije Universiteit Amsterdam, Johns Hopkins, and Ghent University led the field with Bouman and Mullender being among the most productive authors. The United States, together with the Netherlands emerged as dominant research centers with collaborative networks. Most studies were observational and reviews, with a mean evidence level of 3.05 and published primarily in *Journal of Sexual Medicine and Plastic and Reconstructive Surgery*. This first analysis of the 100 most-cited articles in the field of GAS highlights prominent authors, trends in research themes, and dominant geographic regions. Continued comprehensive bibliometric reviews are a necessity to guide future research and clinical practice.

**Level of Evidence**: 5 (Therapeutic)

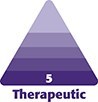

Gender dysphoria is a condition in which a person's gender identity does not match their birth-assigned sex. It is a prevalent medical diagnosis that significantly impacts mental health, social capabilities, and quality of life.^1,2^ Gender-affirming surgeries (GASs) serve as fundamental treatment for gender dysphoria because they improve both health outcomes and life quality for transgender and gender diverse individuals.^1,2^

GAS has experienced significant technical and conceptual development throughout the last century. The field started with experimental techniques and early 20th-century case reports has developed into a complex multidisciplinary field that includes plastic surgery together with urology, gynecology, psychiatry, and endocrinology.^3,4^ Nowadays, GASs include multiple surgical procedures which include facial, chest, and genital operations, for transfeminine and transmasculine patients.^5^

In recent decades, the number of published research on the field has increased substantially because of shifts in clinical practice, policy reforms, insurance coverage expansion, and growing awareness of transgender health requirements.^5,6^

The aim of this study is to identify and evaluate the 100 most-cited articles in the field of GAS. Our bibliometric analysis aims to highlight impactful studies, prominent authors, and institutions, and to explore trends through an analysis of citation patterns, research themes, gender representation, and funding disclosures.

## METHODS

### Search Strategy

We searched the Web of Science (WoS) databases on April 15, 2025, for topics and title words related to GAS. The complete search strategy is detailed in Appendix A. Papers were reviewed by 2 authors. The top 100 articles were obtained according to the citations. We did not apply any restrictions regarding study design or publication year. Eligible articles were sorted by their citation count in descending order. The 100 most-cited articles were selected for detailed analysis. Our main outcome measure was to characterize the 100 most-cited papers related to GAS. The secondary goal of this study was to provide a historical perspective on the most-cited article on the subject. Data recorded is presented in Appendix B.

### Data Extraction

The WoS database contains data on author, journal, country, institution, annual output, funding, and the total number of citations. We used institutional websites, online searches, and name-based assessment by 2 authors (N.Z. and R.S.) to determine the gender of the authors. We excluded 4 articles from our analysis in which the author's gender remained uncertain or unverifiable. Also, we reviewed the abstracts and full texts of the articles to complete the data table comprehensively.

### Bibliometric Analysis and Visualization

Bibliometric analyses were performed using VOSviewer software and the Bibliometrix and Biblioshiny packages for R statistical software. VOSviewer was utilized to create network visualizations in order to illustrate associations among authors, institutions, and keywords, as well as density maps that displayed research activity and thematic concentration. Bibliometrix and Biblioshiny further complemented the analysis by generating longitudinal productivity plots, keyword trend analyses, and network maps depicting collaboration patterns and research clusters. Note, all metadata fields, including author affiliations, funding sources, and contributing organizations, were imported and analyzed without manual editing. As a result, some nodes may represent external organizations or low-frequency contributors. In the network analyses, all nodes are automatically incorporated into clustering algorithms and visual layouts, ensuring the network accurately reflects the underlying data structure.

### Statistical Analysis

Statistical analyses focused on demographic stratification and association measures were conducted using R software (R Core Team, 2013; R Foundation for Statistical Computing, Vienna, Austria; http://www.R-project.org).

## RESULTS

### Scientific Output and Influence

**Figure 1.**
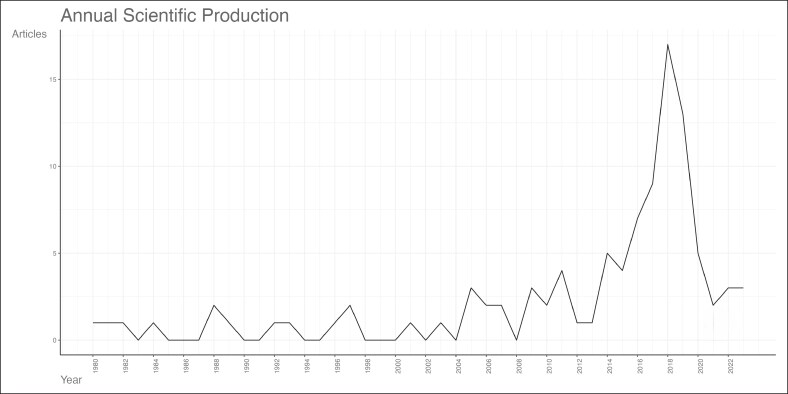
Annual scientific production of scientific publications among the study's cohort. Annual distribution of the number of publications among the top 100 cited articles on gender-affirming surgery. The graph shows the annual trend from 1980 to 2022.

**Figure 2.**
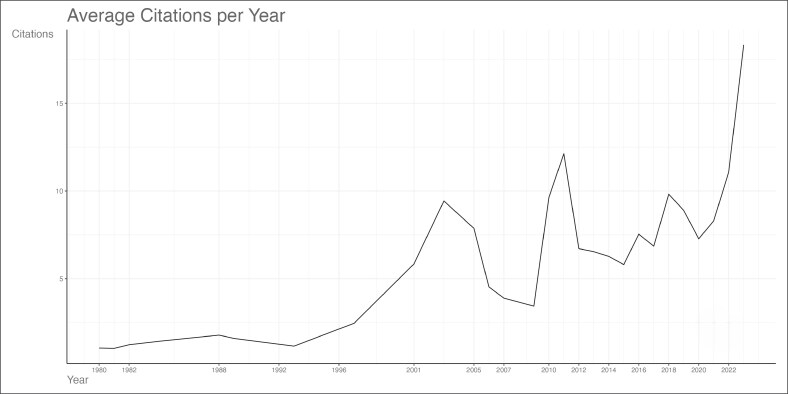
Average citations per year of publications included in the study's cohort. Average number of citations received per publication per year among the top 100 cited articles on gender-affirming surgery. The graph shows the annual trend from 1980 to 2022.

The top 100 articles range from 1980 to 2023, as shown in Figure 1 and Appendix B. The number of citations of the top 100 articles ranged from 36 to 351. The annual scientific production (Figure 1) shows consistent yearly production between 1980 and 2015, before reaching its peak in 2018 and gradually declining thereafter. Citation rates (Figure 2) remained low until the late 1990s, then reached to 2 peaks in 2004 and 2010, followed by sharp increases from 2018 to record levels in 2023-2024, likely because of increased research activities and interest.

**Figure 3.**
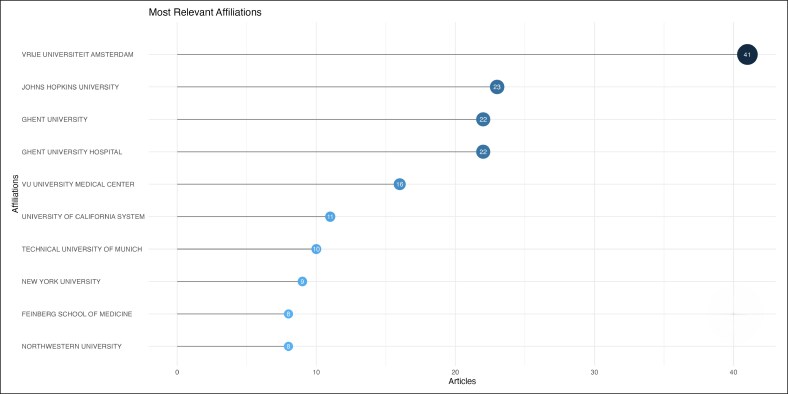
Overall institution productivity of publications included in the study's cohort. The overall productivity of institutions represented in the study cohort, displayed by the number of publications attributed to each institution. The distribution highlights the leading contributing centers.

**Figure 4.**
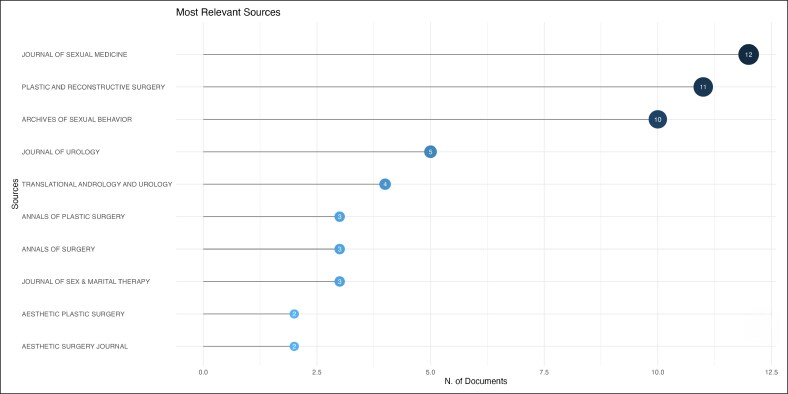
Most relevant sources of publications included in the study's cohort. The overall productivity of journals that published articles in the study cohort, based on the number of included publications attributed to each journal.

**Figure 5.**
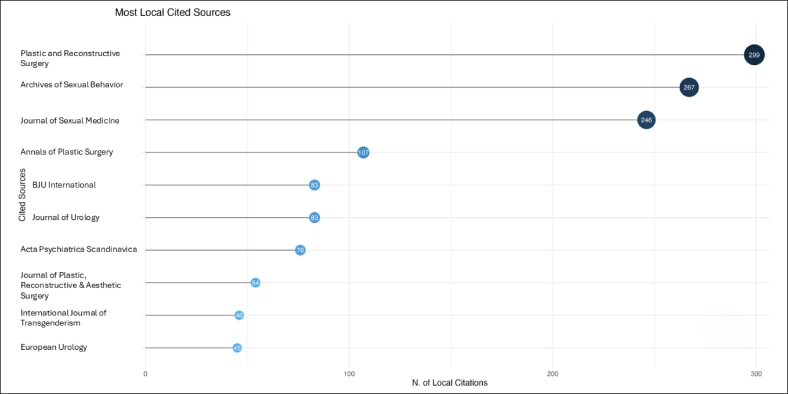
Most locally cited sources of publications included in the study's cohort. The most locally cited sources within the study cohort, based on citation frequency among the included publications.

**Figure 6.**
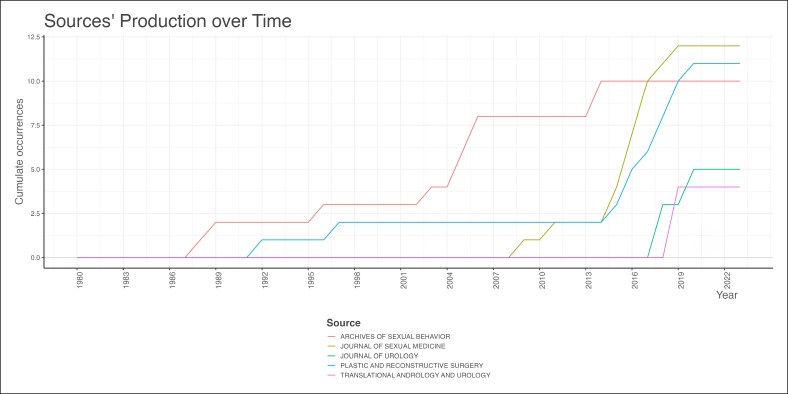
Sources’ production over time of publications included in the study's cohort. The production of sources over time among the publications included in the study cohort. The timeline displays the number of publications contributed by the top 5 sources across the years.

**Figure 7.**
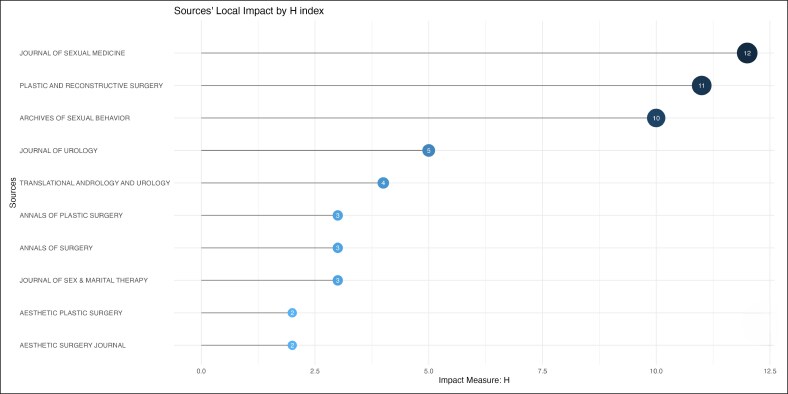
Sources’ local impact by *H*-index of publications included in the study's cohort. This figure presents the local impact of the top 10 sources in the study cohort, measured using the *H*-index calculated from citations within the included publications.

The field is led by Vrije Universiteit Amsterdam, Johns Hopkins University, Ghent University, Ghent University Hospital, which contributed 41, 23, 22, and 22 citations, respectively (Figure 3). The most prominent publication sources are *The Journal of Sexual Medicine* (*n* = 12), followed by *Plastic and Reconstructive Surgery* (*n* = 11), and *Archives of Sexual Behavior* (*n* = 10) publications (Figure 4), with significant publication growth since 2013. These 3 journals also accounted for the most local cited articles (Figure 5), with 246, 299, and 267 citations. Although *Archives of Sexual Behavior* had earlier contributions starting in the 1980s, *Plastic and Reconstructive Surgery*, and *The Journal of Sexual Medicine* published most of their work after 2015 (Figure 6). Their impact was affirmed by their leading *H*-index (*H* = 12, 11, and 10, respectively; Figure 7).

**Figure 8.**
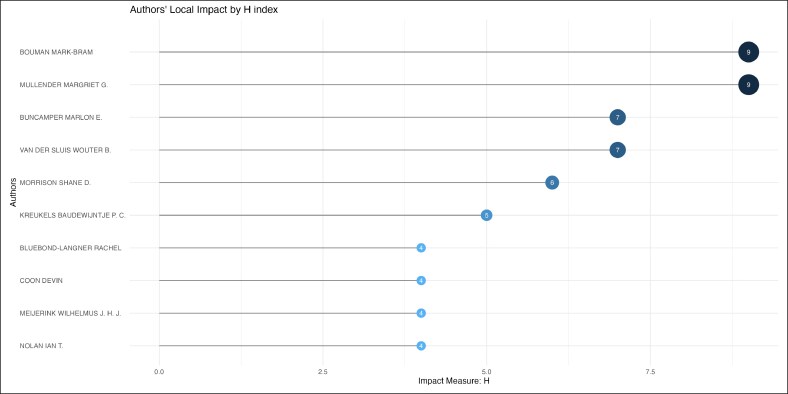
Most relevant authors of publications included in the study's cohort. This figure identifies the top 10 most relevant authors represented in the study cohort, based on the number of included publications attributed to each author.

**Figure 9.**
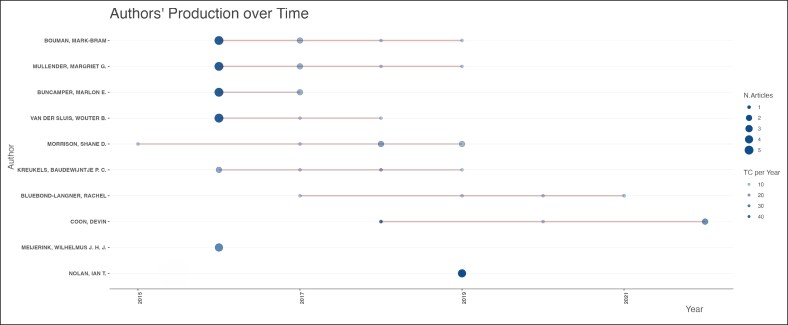
Authors’ production over time of publications included in the study's cohort. This figure illustrates the production of authors over time among the publications included in the study cohort. The timeline shows the number of contributions by key authors across publication years. TC, total citations.

**Figure 10.**
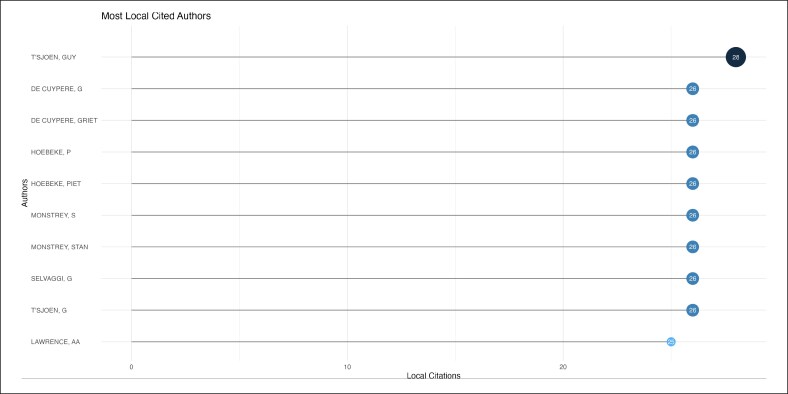
Most locally cited authors of publications included in the study's cohort. The most locally cited authors within the study cohort, based on the number of citations they received from the included publications.

**Figure 11.**
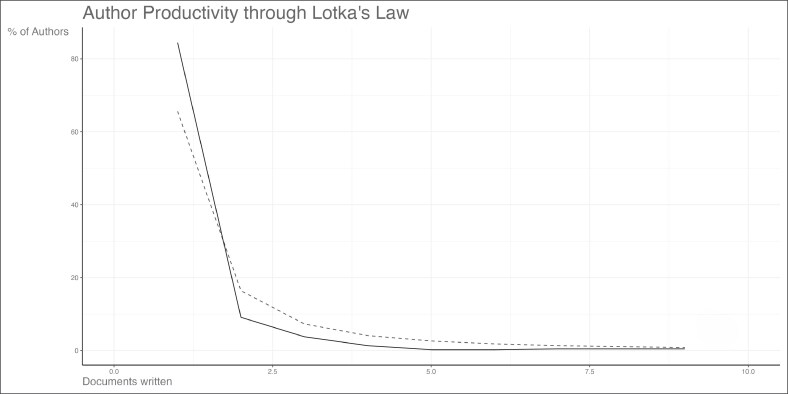
Lotka's law of author productivity of publications included in the study's cohort. This figure illustrates Lotka's law of author productivity for the study cohort, showing the distribution of authors according to the number of publications they contributed.

**Figure 12.**
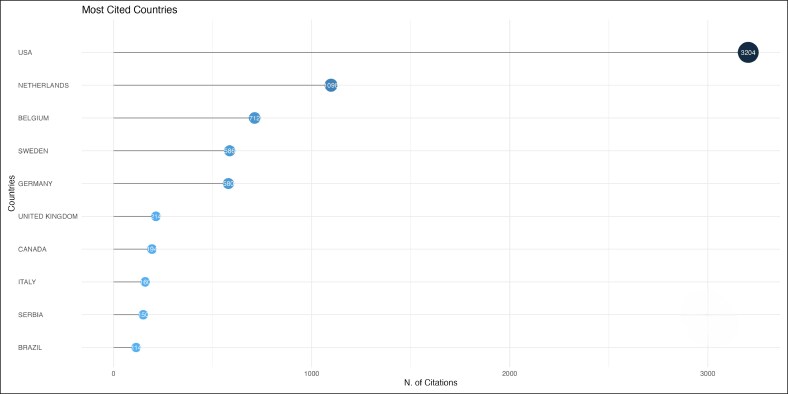
Most-cited countries of publications included in the study's cohort. This figure presents the countries with the highest citation counts among publications in the study cohort.

**Figure 13.**
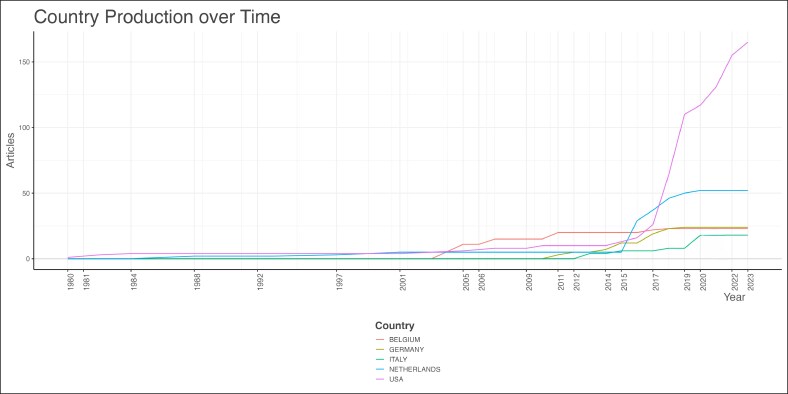
Country scientific production of publications included in the study's cohort. This figure illustrates the scientific production of the top 5 countries represented in the study cohort, based on the number of publications attributed to each country.

Mark-Bram Bouman and Margriet G. Mullender were the top contributing authors, with 9 publications each (Figure 8) during a period from 2016 to 2019 (Figure 9). Local citation analysis (Figure 10) highlighted Guy T'sjoen as the most-cited author with 28 citations, followed by 8 different authors with 26 citations. Author productivity followed Lotka's law (Figure 11), with most contributing only one highly cited article. The United States leads with 3204 citations and has shown growth in article production since 2016. The Netherlands and Belgium follow with 1098 and 712 citations, respectively (Figures 12, 13).

### Thematic and Network Analysis

**Figure 14.**
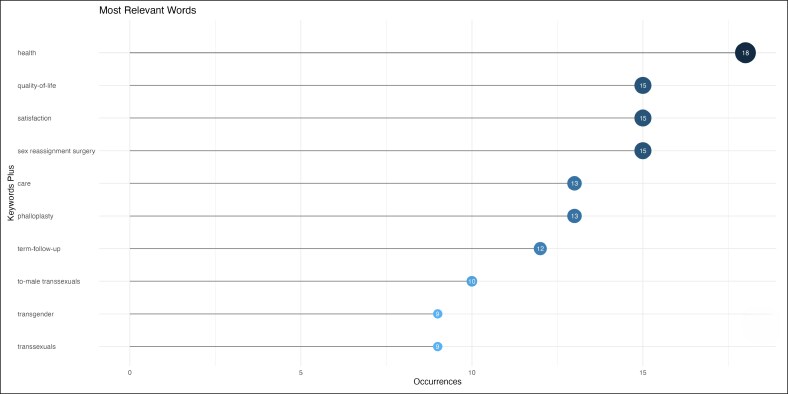
Most relevant keywords of publications included in the study's cohort. This figure presents the most relevant keywords extracted from the publications included in the study cohort, based on their frequency of occurrence.

**Figure 15.**
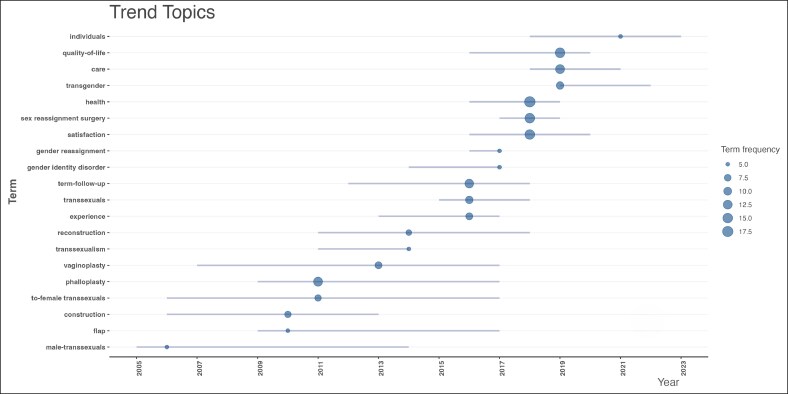
Trend topics of publications included in the study's cohort. This figure illustrates the trending topics within the publications included in the study cohort over time, highlights emerging research themes and shifts in focus within the field across the publication years.

**Figure 16.**
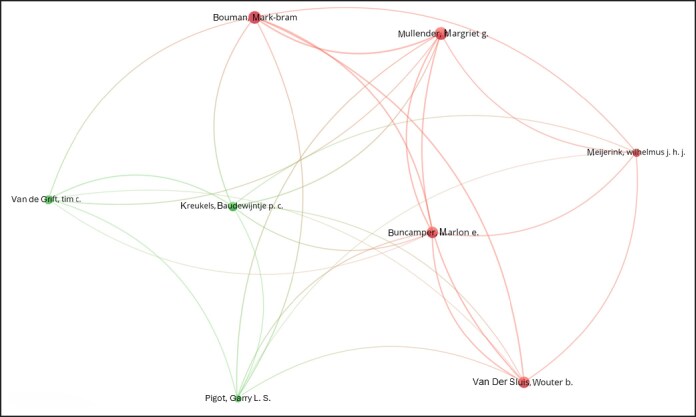
Author collaboration networks of publications included in the study's cohort. This figure presents the author collaboration networks among publications in the study cohort. Nodes represent individual authors, and connections indicate co-authorship relationships. Red and green nodes represent 2 distinct author networks, and lighter nodes act as bridges connecting the clusters, with edges showing co-authorship links.

**Figure 17.**
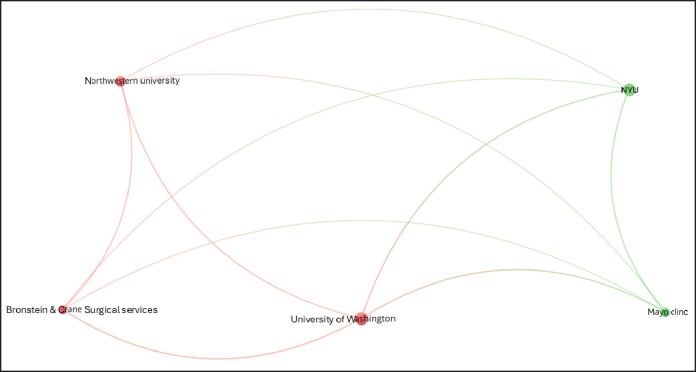
Institutional collaboration networks of publications included in the study's cohort. This figure illustrates institutional collaboration networks among publications in the study cohort. Nodes represent institutions, and connections indicate co-authorship relationships between them. Smaller, low-frequency nodes may represent affiliated research organizations, survey service groups, or external analytics partners documented in publication metadata. Red and green nodes represent 2 distinct institutional collaborative networks, whereas lighter nodes act as bridges connecting the networks, with edges showing co-authorship links.

**Figure 18.**
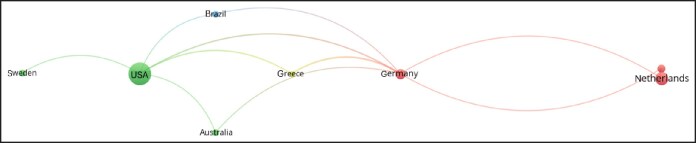
Country-level collaboration networks of publications included in the study's cohort. This figure illustrates country-level collaboration networks among publications in the study cohort. Nodes represent institutions, and connections indicate co-authorship relationships between them. Red and green nodes represent 2 distinct country-level collaborative networks, whereas lighter nodes act as bridges connecting the networks, with edges showing co-authorship links.

Keyword analysis (Figure 14) identified dominant concepts such as “health,” “quality-of-life,” “satisfaction,” “sex reassignment surgery,” and “care.” Trends topics (Figure 15) show “phalloplasty,” “construction,” and “to-female transsexuals” as earlier research concentrations, whereas recent studies increasingly emphasize “satisfaction,” “quality of life,” and “care.” Author collaboration (Figure 16) revealed 2 main clusters centered around 2 Amsterdam-based researchers, Bouman and Kreukels, representing 2 distinct groups: one centered around plastic surgeons and the other around psychologists/sexologists. Institutional collaboration (Figure 17) highlighted 2 main clusters across the United States which include Northwestern University and University of Washington in one, and Mayo Clinic and NYU in the other, with Mayo Clinic functioning as a connection between the clusters. At a national level (Figure 18), the United States and the Netherlands show dominance, with Germany playing a key role in linking them.

### Gender Representation and Sources of Funding

The proportion of female authors was relatively consistent across different author positions, with 35% of first authors, 31% of last authors, and 32% of corresponding authors identified as female. The average number of authors per article was 5.87. The average number of male authors per article was 3.8 (standard deviation [SD] = 3), whereas the average number of females was 2.2 (SD = 2). Seventeen studies received funding. The mean level of evidence was 3.05, reflecting the predominance of observational studies and reviews. Statistically significant results were reported in 45% of the articles.

## DISCUSSION

This bibliometric analysis of the 100 most-cited articles in GAS field reveals valuable insights about the impact, trends, and quality of the current literature. Our findings underscore the growing interest in transgender health in both surgical and nonsurgical aspects. As noted in Appendix B, the most-cited article was “Long-Term Follow-Up of Transsexual Persons Undergoing Sex Reassignment Surgery: Cohort Study in Sweden” by Dhejne et al.^7^ Dhejne et al found that patients who underwent sex reassignment surgery had increased risks of mortality, suicidal behavior, and psychiatric morbidity compared with the general population, indicating that solely surgical procedures may not suffice as treatment for transsexualism and that higher efforts should be conducted to improve psychiatric and somatic care thereafter.^7^

The 100 most-cited articles were published between 1980 and 2023. Diving into the subjects of studies and their primary outcomes provides a comprehensive overview of how GAS has evolved over the years. Early highly cited studies in the 1980s primarily assessed clinical outcomes and patient-centered outcomes, trying to evaluate GAS effectiveness in managing gender dysphoria. This focus continued through the 1990s along with a description of novel surgical techniques for GAS procedures.^8,9^

Likely because of growing interest and acceptance of GAS procedures, the most-cited articles in the 2000s had higher numbers of patients, with a total of 1421 patients included in those articles compared with 795 patients in studies from the 1980s and 1990s (Appendix B). These studies further addressed patient-centered outcomes, along with innovative studies on long-term outcomes.^10-15^ Since 2010, the prominent focus has been on patient-centered outcomes, addressing broader aspects of this field. Although the usual clinical outcomes continued to be assessed, new areas of research emerged, including ethical aspects, insurance coverage, and applications of GAS among adolescents.

Our bibliometric analysis corresponds with the increase in publications and the growing interest in the field as given in Figures 1 and 2, which aligns with the growing social and medical acceptance of transgender health, including significant developments in clinical guidelines and healthcare policy.^16,17^ The release of the seventh version of WPATH Standards of Care in 2012 marked a turning point in the eligibility criteria for hormone therapy and surgery.^18^ The updated guidelines lowered barriers to access care and placed greater emphasis on patient autonomy. Clinical practice evolved through changes in rigid indications that shifted from mandatory real-life experience of gender dysphoria and mental health evaluations to individualized assessment and informed consent.^18^ Additionally, in 2014, the US Department of Health and Human Services allowed Medicare coverage of gender-affirming operations, reversing its longstanding policy.^19^ This evolution likely led to an expanded access to gender-affirming procedures, which boosted publications of research in the years that followed, resulting in a spike in citation rates in 2018 (Figure 2).

We found that research in GAS is primarily dominated by European and American institutions, including Vrije Universiteit Amsterdam, Johns Hopkins University, and Ghent University. The United States leads the field in both scientific output and impact with 3204 total citations and the highest scientific production since 2016. The origins of transgender healthcare emerged during early 20th-century Europe, with developments in both psychological care and surgical procedures.^3^ At the time, patients who wanted gender-affirming procedures often traveled to countries like Germany, where such care was first given. However, the rise of the Nazis and the onset of World War II stopped this early progress, forcing many researchers and clinicians to relocate to the United States.^3^ The migration of researchers and clinicians established the basis for transgender care in the United States which resulted in the establishment of the first gender clinic at Johns Hopkins University in 1966.^20^ The historical trajectory has made the United States and Western Europe as the primary centers shaping the field today. Yet, it highlights the significant research gap in low- and middle-income countries and regions with limited access to gender-affirming care, underscoring the need for international collaboration through shared training and knowledge.^21^

Keyword analysis and thematic mapping indicate a change in the field's focus. Although early literature concentrated on surgical techniques and outcomes (highlighting words such as “phalloplasty” and “construction”), recent studies primarily focused on patient-centered outcomes (with words such as “satisfaction,” “quality of life,” and “care”). This shift reflects a broadening in research focus, from prioritizing surgical techniques and technical outcomes of reconstruction to exploring the personal experiences and subjective outcomes of transgender patients. It also corresponds with a growing acknowledgment of the diversity within transgender communities.^18,22^

In addition, a brief review of the abstracts of the most cited suggests that the types of GAS have evolved over time. In the 1980s, all the studies were generally addressed to GAS without specifying a certain procedure. Through the 2000s studies have reviewed novel surgical techniques for GAS procedures and were more “procedure specific,” such as clitoroplasty, phalloplasty, vaginoplasty, and metoidioplasty.^8,9,14^ Figure 15 further highlights phalloplasty and vaginoplasty as a main trend in this timeframe. After 2010, corresponding with the evolution in the field, we observed a broadened surgical spectrum expanding to top surgeries, facial feminization/musculation, penectomy, and orchiectomy, reflecting both expansion in available procedures and increasing interest across multiple components of transition.

As shown in Figure 11, author productivity is consistent with the principle of Lotka's law, stating that there is an inverse relationship between the number of authors and the number of their publication. This pattern highlights the presence of a core group of highly productive authors. Network analysis identifies 2 leading collaborative groups as the most productive contributors. The first group consists exclusively of plastic surgeons, including surgeons such as Bouman, Mullender, Buncamper, and van der Sluis, whereas the second group consists primarily of mental health professionals, such as Kreukels, Pigot, and Van De Grift. Together, these networks illustrate the multidisciplinary nature of this field, which combines surgical with mental healthcare to address the complex needs of transgender patients.

The majority of the top 100 most-cited articles were observational studies or review, with a mean evidence level of 3.05. Clinically, the low level of evidence reflects the notable shortage of randomized controlled studies. GAS research would greatly benefit from multi-center collaborations focused on validating procedures and evaluating both clinical and psychological long-term outcomes.

The study's findings about female representation are consistent with the proportion of females reported in plastic surgery residency programs across the United States.^23^ Females accounted for 35% of first authors, 31% of last authors, and 32% of corresponding authors, aligning with the 32.5% residency rate reported in 2014.^23^ However, this percentage of female first authors in the field of GAS is considerably higher than what has been reported in the general plastic surgery literature.^23^

This study has several limitations. First, the number of citations provided by the WoS may not obtain all relevant publications. Other search methods may have produced a different list of the most-cited articles in the field of GAS. Although the WoS is known to be a trusted publisher-independent global citation database, using multiple search engines could enable a better validation of the top 100 most-cited articles list. Second, examining the number of citations of an article is a limited and objective measure of the relevance of a study. Third, our analysis does not include recently published studies, as they may not have generated a large number of citations yet, as they have only recently been published. Moreover, we determined the gender of the authors using online sources such as institutional websites and photographs, along with our own assessment, based on their names. This may have led to potential misclassification or the exclusion of certain authors. Finally, because we focused only on the 100 cited articles, we may have overlooked broader trends and certain areas of interest. To address this, future bibliometric studies should consider a broader range of articles, beyond the most-cited ones.

## CONCLUSIONS

In conclusion, to our knowledge, this is the first report of the top-cited articles on GAS. Our findings highlight key researchers, emerging thematic trends, and geographic concentrations. Because this field rapidly expands to encompass a broader range of surgical techniques and quality-of-life assessments, ongoing and more comprehensive bibliometric reviews will be essential to guide future research and support the alignment of clinical guidelines worldwide.

## Supplemental Material

This article contains supplemental material located online at https://doi.org/10.1093/asjof/ojag020.

## Supplementary Material

ojag020_Supplementary_Data
