# Supplementary material for: Bibliometric Analysis of the 100 Most-Cited Publications in Gender-Affirming Surgery
Source: Aesthet Surg J Open Forum. 2026 Feb 4;8:ojag020. doi: 10.1093/asjof/ojag020 (PMC12968774; doi:10.1093/asjof/ojag020)
Supplement: ojag020_Supplementary_Data [file ojag020_supplementary_data.zip › Appendix A.docx]

**Appendix A**. Search Strategy

TI=(("transgender" AND ("top surgery" OR "augmentation mammaplasty" OR "mastectomy" OR "breast augmentation" OR "phalloplasty" OR "metoidioplasty" OR "vaginoplasty" OR "vulvoplasty" OR "orchiectomy" OR "penectomy" OR "facial feminization" OR "facial masculinization" OR "Adam’s apple reduction" OR "tracheal shave"))

OR ("gender-affirming surgery")

OR ("gender-confirming surgery")

OR ("sex reassignment surgery")

OR ("gender reassignment surgery")

OR (("gender affirming" OR "gender-confirming") AND ("surgery" OR "procedure" OR "plastic surgery"))

OR ("nonbinary" AND "surgery")

OR ("gender diverse" AND "surgery"))
