# Supplementary material for: Bibliometric Analysis of the 100 Most-Cited Publications in Gender-Affirming Surgery
Source: Aesthet Surg J Open Forum. 2026 Feb 4;8:ojag020. doi: 10.1093/asjof/ojag020 (PMC12968774; doi:10.1093/asjof/ojag020)
Supplement: ojag020_Supplementary_Data [file ojag020_supplementary_data.zip › Appendix B.pdf]

Serial	First Author	N Authors	Cor	Corr	Gender Fir	Gender Las	Gender C	N Authors Male	N Autors Fema	Country First Auth	Country Last A	Level of Evidence	Type of Study	Year of Publi	Funding (Yes/No)	Type of Funding	Subject of Study	Outcome (Primary)	Outcome (Secondary)	Number of Patients	Results Statistically Significant?
1	Dhejne, Cecilia	6	y		f	m	f	4	2	Sweden	Sweden	3	Retrospective St	2011	y	Stockholm Count	comparison of m	Overall mortality after s	Suicide, suicide attempt	324	Y
2	Canner, Joseph K.	8		y	m	m	m	6	2	USA	USA	3	Retrospective St	2018	y	Patient-Centered	trends of GAS in U	Incidence of gender-af	Patient demographics, i	4,118	y
3	De Cuypere, G	9	y		f	m	f	6	3	Belgium	Belgium	2	Prospective Coh	2005			Long-term sexual	Sexual health and satis	Emotional and social ful	55	y
4	Ainsworth, Tiffiny A.	2		y	f	m	m	1	1	USA	USA	3	Cross-sectional	2010			Impact of facial fe	Mental health-related	Comparison of mental h	247	y
5	Lawrence, AA	1	y		f		f	0	1	USA		3	Cross-sectional	2003			Factors linked to	Patient satisfaction wi	Associations between s	232	n
6	Nolan, Ian T.	3		y	m	f	f	2	1	USA		1	systematic revie	2019			temporal trends i	Prevalence and demog	NR	x	n
7	Wierckx, Katrien	11	y		f	m	f	5	6	Belgium	Belgium	3	Cross-sectional	2011			Quality of life and	Self-reported physical	frequency of masturbati	49	y
8	Dhejne, Cecilia	4	y		f	m	f	2	2	Sweden	Sweden	3	Retrospective St	2014			Incidence and pre	Incidence, prevalence	Trends in applications o	767	n
9	Selvaggi, Gennaro	2		y	m	m	m	2	0	UK	UK	5	Narrative review	2011			Overview of genit	Description of availabl	Guidelines for eligibility,	x	n
10	Smith, YLS	3		y	f	f	f	0	3	Netherlands	Netherlands	2	Prospective Follo	2001			Outcomes in adol	Psychological, social,	Pre- and post-treatment	47	NA
11	Buncamper, Marlon I	8	y		m	f	m	5	3	Netherlands	Netherlands	3	Retrospective St	2016			Surgical outcome	Intraoperative and pos	Risk factors for complic	475	y
12	van de Grift, Tim C.	5		y	m	f	f	1	4	Netherlands	Netherlands	3	Cross-sectional	2018			Association betw	Postoperative satisfac	Self-reported surgical c	201	y
13	Lawrence, AA	1	y		f		f	0	1	USA		3	Cross-sectional	2006			complications an	surgical complications	Association of individua	232	y
14	Lawrence, AA	1	y		f		f	0	1	USA		3	Cross-sectional	2005			Changes in sexua	Sexual orientation, nu	autogynephilia, mastur	232	y
15	Selvaggi, Gennaro	6	y		m	m	m	5	1	Belgium	Belgium	3	Cross-sectional,	2007			Genital tactile an	Tactile and erogenous	Orgasmic capacity, surg	57	n
16	Mahfouda, Simone	7		y	f	m	m	3	4	Australia	Germany	5	Narrative review	2019	y	University Postgr	Overview of the o	mental health, cogniti	Side effects, quality of li	x	n
17	Gaither, Thomas W.	7		y	m	m	m	5	2	USA	USA	3	Retrospective St	2018			complications fol	Postoperative complic	Risk factor analysis (age	330	y
18	KUIPER, B	2		y	m	f	f	1	1	Netherlands	Netherlands	3	Retrospective St	1988	y	NOAPR (Netherla	Therapeutic effec	Self-reported satisfact	Comparison between or	141	n
19	Neto, R. Rossi	5	y		m	NR	m	NR	NR	Germany	Germany	3	Retrospective St	2012			Surgical outcome	Incidence and type of s	Functional outcomes, n	332	n
20	Ascha, Mona	5		y	f	m	m	4	1	USA	USA	3	Retrospective St	2018			Comparison of ur	Urethral complication	Association of patient fa	213	y
21	Hess, Jochen	5	y		m	m	m	5	0	Brazil	Brazil	3	Retrospective St	2014	y	AMS American Me	Patient overall sa	Satisfaction with life a	Satisfaction with outwa	119	y
22	Hoebeke, P	8	y		m	m	m	7	1	Belgium	Belgium	3	Cross-sectional	2005			Impact of Sex Rea	Voiding habits and low	Uro-flowmetry data, inci	55	n
23	Bouman, Mark-Bram	7	y		m	f	m	4	3	Netherlands	Netherlands	3	Cross-sectional	2016			Patient-reported	Patient-reported funct	Quality of life, satisfacti	31	y
24	Costantino, Antoniet	6		y	f	f	f	2	4	italy	italy	2	Prospective Coh	2013			Sexual function a	Sexual function and m	reproductive hormone l	50	y
25	Wright, Jason D.	5	y		m	f	m	3	2	United States	United States	3	Retrospective St	2023			Trends in inpatier	Annual number of inpa	Temporal trends in types	48019	n
26	van der Sluis, Woute	9	y		m	f	m	6	3	Netherlands	Netherlands	3	Retrospective St	2016			Long-term surgic	Surgical complications	Functional and aestheti	24	n
27	Schneider, Florian	9		y	m	f	f	6	3	Germany	Germany	3	Retrospective St	2015	y	DFG-Researuch U	Effects of differen	Blood hormone and int	Evaluation of testicular t	108	y
28	Eldh, J	3	y		m	f	m	1	2	Sweden	Sweden	2	Prospective Coh	1997			long-term outcon	Surgical outcome, Soc	personal and social stab	136	NA
29	Papadopulos, Nikola	10	y		m	m	m	9	1	Greece	Germany	3	Cross-sectional	2017			Quality of life and	Quality of life after SRS	Satisfaction with body i	47	y
30	McEvenue, Giancarl	4	y		m	m	m	3	1	Canada	Canada	3	Retrospective St	2018			Safety and aesthe	safety profile and aest	Complication and reope	679	y
31	van de Grift, Tim C.	12	y		m	f	m	7	5	Netherlands	Netherlands	3	Cross-sectional	2017			motivations for a	Motivations for surgery	voiding issues, complic	21	y
32	Kailas, Maya	4		y	f	m	m	2	2	USA	USA	3	Cross-sectional	2017			Prevalence and ty	Prevalence and type of	differences between tra	99	NA
33	Morrison, Shane D.	6			m	f	m	4	2	USA	USA	4	Retrospective St	2015			Long-Term Outco	complication rates, re	Patient satisfaction with	47	y
34	El-Hadi, Hadal	4	y		f	m	f	1	3	Canada and Unite	Canada and Un	3	Cross-sectional	2018			Perceived satisfa	Perceived satisfaction	Barriers to care (financi	32	n
35	Kuhn, Annette	6	y		f	m	f	4	2	Switzerland	Switzerland	3	Case-control stu	2009			Quality of life and	Quality of life (King's H	Satisfaction, Emotions,	55	y
36	Morrison, Shane D.	3		y	m	m	m	3	0	USA	USA	5	Narrative review	2017			Overview of surgi	Overview of FtM surgic	Complications (especia	x	n
37	Bluebond-Langner, F	6	y		f	f	f	3	3	USA	USA	3	Retrospective St	2017			Evaluation of surg	Rate of aesthetic revisi	Postoperative complica	295	y
38	Bouman, Mark-Bram	6	y		m	m	m	4	2	Netherlands	Netherlands	2	Prospective Coh	2016			Surgical outcome	Intraoperative and pos	hospitalization duration	42	n
39	Falcone, Marco	6		y	m	m	m	5	1	UK	UK	3	Retrospective St	2018			Outcomes of infla	Complication and revis	Patient/partner satisfac	247	y
40	Badolucci, Constanz	8			f	m	f	3	5	Spain	Spain	3	Cross-sectional	2015			Perception of sex	Sexual quality of life sc	Factors associated with	103	y
41	Rakic, Z	4	y		m	f	m	3	1	Serbia	Serbia	3	Cross-sectional	1996			Quality of life out	Quality of life assessm	Body attitude, relations	32	n
42	Veerman, H.	8	y		NR	NR	NR	NR	NR	Netherlands	Netherlands	3	Retrospective St	2020			Urological compl	Urological complicatio	Stricture and fistula for	63	NA
43	da Silva, Dhiordan C	10	y		m	f	m	5	5	Brazil	Brazil	2	Prospective Coh	2016	y	Coordination for t	Impact of sex rea	Quality of Life Assessm	Impact of additional sur	47	y
44	Hage, JJ	4	y		m	m	m	4	0	Netherlands	Netherlands	5	Narrative review	1997			Considerations a	Identification and mod	Surgical planning consi	x	N
45	Djordjevic, Miroslav I	9	y		m	m	m	7	2	Serbia	Serbia	3	Retrospective St	2009	y	Republic Ministry	Outcomes and te	Patient satisfaction re	Urethral complications,	82	n
46	Dubov, Alex	2	y		m	f	m	1	1	USA	USA	5	Narrative review	2018	y	National Institute	Ethical considera	Access to facial femini	facial feminization surg	x	n
47	LUNDSTROM, B	3	NR	NR	NR	NR		NR	NR	NR	NR	5	Narrative review	1984			Outcomes of sex	Failure rate of sex reas	Risk factors for poor out	x	N
48	Zavlin, Dmitry	10		y	m	m	m	10	0	Germany	Germany	2	Prospective Coh	2018			Patient satisfacti	Patient-reported satisf	Improvement rates in ev	40	y
49	ROSS, MW	2	y		m	m	m	2	0	Austrelia	Austrelia	3	Retrospective St	1989			Relationship betv	Postoperative psychop	Factors influencing post	14	y
50	Wernick, Jeremy A.	5	y		m	m	m	3	2	USA	USA	1	Systematic Revie	2019			Systematic review	Psychological benefits	x	x	n
51	Remington, Austin C	9		y	m	m	m	8	1	USA	USA	1	Systematic Revie	2018			comparison of ph	Urethral and flap comp	Voiding ability, sexual fu	1351	y
52	Ammari, Tareq	4		y	m	m	m	2	2	USA	USA	5	Literature review	2019			Evaluation of surg	Complication rates an	Decision algorithms to d	x	n
53	Jiang, David	4		y	m	m	m	4	0	USA	USA	3	Retrospective St	2018			Factors influenci	Reasons for choosing	Demographics (age, BM	486	y
54	van der Sluis, Woute	7	y		m	m	m	7	1	Netherlands	Netherlands	4	Case series	2017			Outcomes of dou	Intraoperative and pos	flap sizes, hospitalizatio	19	n
55	Oles, Norah	10		y	f	m	m	6	4	USA	USA	1	Systematic Revie	2022			Systematic review	Types and rates of com	Frequency and validatio	NR	N
56	Weissler, Jason M.	7		y	m	f	f	6	1	USA	USA	5	Review	2018			Framework and c	Surgical outcomes, Ps	presenting multidiscipli	x	n
57	LOTHSTEIN, LM	1	y		m		m	1	0	NR	NR	5	Review article	1982			overview of histor	overall success rates o	Role of psychotherapy i	x	n
58	van der Sluis, Woute	6			m	m	m	5	1	Netherlands	Netherlands	3	Retrospective St	2016			management of n	Incidence and manage	Risk factors for fistula d	1082	y
59	Ines Lobato, Maria In	9	y		f	m	f	3	6	Brazil		3	Cross-sectional	2006			the effect of sex r	Satisfaction with sexu	partnerships, and family	19	n
60	Chen, Mang L.	4		y	m	f	f	1	3	USA	USA	5	Narrative review	2019			Overview and des	Types and principles o	perioperative considera	x	n
61	Al-Tamimi, Muhamm	7		y	m	m	m	6	1	Netherlands	Netherlands	3	Retrospective St	2018			The effect of colp	Incidence of urethral fi	Fistula closure rate after	294	y
62	LeBreton, Marianne	7		y	f	m	m	5	2	Canada	Canada	2	Prospective Coh	2017			Genital sensory d	genital sensory detecti	Frequency and type of s	28	y
63	Kim, Seok-Kwun	5		y	m	m	m	5	0	Korea	Korea	3	Retrospective St	2010			presenting a new	Incidence of urethrocu	Flap loss, incidence of u	70	n
64	HUNT, DD	2	NR	NR	m	m	NR	2	0	USA	USA	2	Prospective Coh	1980			Long-term psych	Changes in psychopat	Partner and family persp	17	NA
65	Ngaage, Ledibabari N	7		y	m	f	f	2	5	USA	USA	3	Cross-sectional	2019			Assessment of in	Insurance coverage rat	Alignment of insurance	57	y
66	Dy, Geolani W.	4	y		f	m	f	3	1	USA	USA	1	Systematic Revie	2019			describe existing	Types of PROMs and Q	Strengths and limitation	x	n
67	Boas, Samuel R.	8		y	m	m	m	7	1	USA	USA	3	Retrospective St	2019			Outcomes and pr	Rate of revision surger	Postoperative complica	117	y
68	Defreyne, Justine	3	y		f	m	f	2	1	Belgium	Belgium	1	Literature Review	2017			Review of healthc	Quality of life and psyc	Satisfaction rates with g	x	NR
69	Gomez-Gil, Esther	5	y		f	m	f	2	3	Spain	Spain	3	Cross-sectional	2014	y	Spanish Ministeri	Determinants of	pre-surgery Quality of	Associations between Q	193	y
70	PAULY, IB	1	y		m		m	1	0	USA		3	Retrospective St	1981			Outcomes of sex	Updated evaluation of	present the psychothera	366	NA
71	Vukadinovic, Vojkan	4			m	m	m	4	0	Serbia	Serbia	3	Retrospective St	2014			The role of clitora	Aesthetic, functional,	postoperative complica	97	n
72	Santucci, Richard A.	1	y		m		m	1	0	USA		5	Literature review	2018			to present urethr	Urethral Complication	Success and failure rate	x	n
73	Oles, Norah	10		y	f	m	m	5	5	USA	USA	1	Systematic Revie	2022			review of publish	Patient-centered outc	proportion of studies us	x	n
74	Swan, Jaime	6		y	f	f	f	2	4	Australia	Australia	1	Systematic Revie	2023			Systematic review	Changes in mental hea	Quality of life and life sa	x	n
75	Djordjevic, M. L.	9	y		m	m	m	9	0	Serbia	Serbia	2	Prospective Coh	2009	y	Ministry of Scienc	To intoduce a tec	neophallic length, neo	Urinary function issues	38	n
76	FANG, RH	3	NR	NR	m	m		3	0	Taiwan	Taiwan	4	Case series	1992			To introduce a ne	Survival of neoclitoris,	urine leakage rates, sex	9	n
77	Kozato, Aki	10		y	f	m	m	6	4	USA	USA	3	Retrospective St	2021			Risk of venous th	Incidence of venous th	The effect of perioperati	919	n
78	Ngaage, Ledibabari N	8		y	f	f	f	2	6	USA	USA	3	Cross-sectional	2020			Insurance covera	Proportion of insurers	Variability in specific co	57	n
79	Pariser, Joseph J.	2	y		m	m	m	2	0	USA	USA	5	Narrative review	2019			Description of pe	Description of surgical	Review of literature on p	x	n
80	Narayan, Sasha Kara	11		y	f	m	m	5	6	USA	USA	5	Literature Review	2021			Classification of r	Classification and freq	a literature review on the	NR	n
81	van de Grift, Tim C.	5	y		m	f	m	3	2	Netherland	Netherland	2	Prospective Coh	2019			Sexual activity an	Sexual activity status p	insights into genital sen	38	n
82	Papadopulos, Nikola	9	y		m	m	m	9	9	Taiwan	Taiwan	2	Prospective Coh	2017			Outcomes of a m	vaginal depth and geni	postoperative complica	40	n
83	Martinson, Tyler G.	5		y	m	m	m	2	3	USA	USA	3	Retrospective St	2020			Impact of high bo	Prevalence of obesity a	Effectiveness of a self-m	1457	n
84	Pan, Shu	2		y	m	m	m	2	0	USA	USA	5	Literature review	2018			Overview of curre	types and techniques	complications of GAS su	x	n
85	Giami, Alain	2	y		m	m	m	2	0	France	France	3	Cross-sectional	2014			overview of the cu	Prevalence and role of	Sociodemographic char	381	y
86	Kidd, Jeremy D.	3	y		m	m	m	3	0	USA	USA	3	Cross-sectional	2018	y	National Institute	Relationship betv	Prevalence and patter	Age of smoking onset, fr	1104	y
87	Lee, Hyemin	5		y	f	m	m	1	4	South Korea	South Korea	3	Cross-sectional	2018	y	Daum storyfundir	Experiences and	Prevalence of GID diag	Prevalence of barriers, s	278	y
88	Ellent, Elizabeth	2		y	f	m	m	1	1	USA	USA	4	Case report	2016	y	Merck; Genetech;	presenting a case	Diagnosis and manage	x	1	n
89	Castellano, E.	9	y		NR	NR	NR	NR	NR	Italy	Italy	3	Cross-sectional	2015			The relationship	Quality of life and queli	Relationship between h	120	y
90	Caprini, Rachel M.	10		y	f	f	f	3	7	USA	USA	3	Cross sectional s	2023	y	Bernard G. Sarnat	The effect of gend	Changes in psychosoc	the effects of other gend	169	y
91	Levy, Jason A.	8	y		m	f	m	5	3	USA	USA	3	Retrospective St	2019			outcomes, compl	Surgical outcomes an	Risk factors for complic	240	n
92	RUBIN, SO	1	y		f		f	0	1	Sweden		4	Case series	1993			outcomes and a r	Success rate of a modi	Complications (necrosi	13	n
93	Rooker, Steven A.	6	y		m	m	m	4	2	USA	USA	1	Meta analysis	2019			review of outcom	Rates and types of com	Prosthesis type, patient	1056	n
94	Hoenig, Johannes Fr	1	y		m		m	1	0	Germany	Germany	4	Case series	2011			clinical outcome	Clinical outcomes and	Surgical ease, volume of	21	n
95	BOUMAN, FG	1	y		m		m	1	0	Netherlands		4	Case series	1988			outcomes of mal	Patient-reported satisf	surgical regret rate, nee	67	NA
96	Ascha, Mona	12		y	f	f	f	3	9	USA	USA	2	Prospective Coh	2022	y	National Institute	impact of mastec	Chest Dysphoria Meas	Gender congruence, Bo	81	y
97	Vedovo, Francesca	22	y		f	m	f	18	4	Italy	Italy	4	Prospective stuf	2020			Development and	Psychometric reliabilit	Test-retest reliability an	122	NR
98	Kukreja, Promil	5	y		m	m	m	4	1	USA	USA	4	Case report	2020			describe the use	Postoperative pain sco	comparison of the new t	1	n
99	Stowell, Justin T.	16	y		m	f	m	7	9	USA	USA	5	Literature review	2019	y	GE Healthcare gra	review of the vari	imaging appearance of	Detection and character	x	n
100	Papadopulos, Nikola	9	y		m	m	m	9	0	Germany	Germany	4	Prospective Coh	2017			Evaluation of mal	Changes in quality of li	The effect of SRS on part	39	y
